# The Modified JiuWei QiangHuo Decoction Alleviated Severe Lung Injury Induced by H1N1 Influenza Virus by Regulating the NF-***κ***B Pathway in Mice

**DOI:** 10.1155/2015/790739

**Published:** 2015-05-18

**Authors:** Lijuan Chen, Xin Yan, Qianlin Yan, Jiajun Fan, Hai Huang, Xunlong Shi, Lei Han, Tianxiong Han, Haiyan Zhu

**Affiliations:** ^1^Department of Chinese Medicine, Shanghai Tenth People's Hospital, Tongji University School of Medicine, Shanghai 200092, China; ^2^Institute of Chinese Medicine, Tongji University School of Medicine, Shanghai 200092, China; ^3^Department of Biosynthesis, School of Pharmacy, Fudan University, 826 Zhangheng Road, Shanghai 201203, China

## Abstract

A new approach to treat infections of highly pathogenic influenza virus is to inhibit excessive innate immune response. JiuWei QiangHuo decoction has been used for centuries for the treatment of pulmonary disorders in China. In this study, we evaluated the anti-inflammatory activities of the modified JiuWei QiangHuo (MJWQH) decoction in the treatment of influenza A (H1N1) virus-induced severe pneumonia in mice. The results showed that MJWQH significantly increased the survival rate of H1N1-infected mice and suppressed the production of TNF-*α*, IL-1, IL-6, MCP-1, RANTES, and IFN-*α* on day 4 after infection. Moreover, oral administration of MJWQH efficiently inhibited virus replication and alleviated the severity of lung injuries. The results also showed that MJWQH may have potential therapeutic effect on severe lung injury induced by H1N1 virus by regulating the NF-*κ*B pathway. Our study suggested that MJWQH might be an alternative therapy for the treatment of viral pneumonia.

## 1. Introduction

Influenza A (H1N1) virus can cause severe respiratory diseases and lead to pandemic outbreaks in humans. Some severe patients may develop acute lung injury and even acute respiratory distress syndrome, which are the most common causes of death in these H1N1-infected patients [1]. However, influenza continues to evolve and antiviral drugs are usually not given in the early stage of virus infection, thus rendering the antiviral therapy ineffective. It is suggested that modulation of the host immune response has the potential advantage of exerting less-selective pressure on viral populations [2]. Many traditional Chinese medicines (TCM) and herbs have been shown to have immunomodulatory, anti-inflammatory, antiviral, and antioxidant activities [3–5]. There are a number of clinical trials evaluating the potential benefit of TCM in the treatment of H1N1-induced pneumonia [6]. It seems to be a promising approach to extract effective ingredients from TCM for the treatment of influenza virus-induced severe pneumonia.

The recruitment of innate immune cells into the lung and excessive proinflammatory cytokines and chemokines are hallmarks of influenza virus infection [7]. It has been shown that early recruitment of inflammatory leukocytes to the lung, followed by excessive early cytokine responses, is the key contributor to the morbidity and mortality of H1N1 virus infections [8, 9]. Toll-like receptors (TLRs) are the main intracellular/extracellular immune cell receptors that recognize pathogen-associated molecular patterns of the microbes and foreign particles, including viruses, to induce several immune cell functions ranging from migration and phagocytosis to inflammatory cytokine expression [10–13]. TLR7 is located in the membranes of the endosomal compartment and can recognize viral single-stranded RNA (ssRNA) [14]. TLR7 expression is upregulated in pulmonary epithelial cells and macrophages after virus infection. Upon stimulation, cells recognize viral ssRNA via TLR7 and activate downstream signaling molecules through a MyD88 dependent pathway, causing subsequent cytokine storm and triggering inflammatory lung injury [15, 16].

JiuWei QiangHuo (JWQH) decoction was first introduced in the monograph* Cishinanzhi *written by an influential TCM physician Zhang Yuansu of Jin Dynasty (1151–1234). It has been used for over 900 years for the treatment of pulmonary disorders, such as common cold, bronchial infections, and pneumonia. This decoction has recently been modified by Dr. Yan Dexin, a Grandmaster of Chinese Medicine granted by the Ministry of Health of China, which consists of eight herbs including* Rhizoma et Radix Notopterygii, Herba Taraxaci, Radix Scutellariae, Radix Astragali, Rhizoma Atractylodis, Radix Saposhnikoviae, Herba Houttuyniae, *and* Radix Trichosanthis.* The modified JWQH (MJWQH) has been shown to be effective in patients with severe pneumonia induced by influenza virus. It may reverse H1N1-induced pneumonia in mice by regulating the metabolism of arachidonic acid, fatty acid, and amino acid and is thus involved in anti-inflammatory activity and cell protection [17]. Based on previous research findings and the pathogenesis of H1N1-induced pneumonia, we hypothesize that MJWQH has an immunoregulatory effect in the early stages of the disease by inhibiting excessive immune response, thus inhibiting virus replication, lung inflammation, and severe lung injury.

## 2. Materials and Methods

### 2.1. Preparation of MJWQH

MJWQH was prepared from eight dried raw materials (Table 1) purchased from Shanghai Shuguang Hospital (Shanghai, China) and authenticated by a physician in Fudan University. Voucher specimens were deposited in the Herbarium Center of the Department of Pharmacognosy, School of Pharmacy, Fudan University. All raw materials were extracted by boiling in distilled water (about 6-fold the weight of the mixture) at 100°C for 20 min and then filtered. The filtrates from two decoctions were combined, and the decoction was dried in vacuo at 70°C and ground into powder for use. The yield of the extraction was 24%. The extracts were stored in an airtight container at −80°C until further analysis.

### 2.2. Chromatographic Analysis of MJWQH

**Figure 1:**
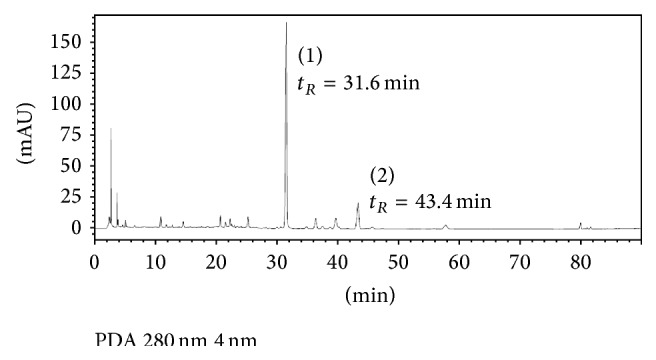
HPLC chromatogram of MJWQH (280 nm). Calycosin-7-*O*-*β*-D-glucopyranoside (1) and baicalin (2) were separated from MJWQH at a retention time of 31.6 and 43.4 min, respectively.

MJWQH was dissolved in pyrogen-free isotonic saline and filtered through a 0.2 mm filter (Microgen, Laguna Hills, CA, USA) before high performance liquid chromatography (HPLC) analysis (Figure 1). HPLC analysis was performed on an Agilent series 1100 HPLC system (Agilent, Waldbronn, Germany) equipped with quaternary pump, diode-array detector, autosampler, and column compartment. Calycosin-7-O-*β*-D-glucopyranoside and baicalin purchased from the National Institute for the Control of Pharmaceutical and Biological Products (Beijing, China) were used as the external standards in HPLC. Chromatographic separation was performed on a Zorbax XDB-C18 column (5 *μ*m, *φ*  4.6 × 250 mm, Agilent Technologies, USA). The mobile phase consisted of acetonitrile (A) and water containing 0.2% acetic acid (B), and the following gradient program was used: 5% A in the first 10 min, then a linear gradient to 36% A over 60 min, and then a linear gradient to 85% A over 30 min. The mobile phase flow rate was 0.8 mL/min, the detector was monitored at 280 nm, the spectral data for all peaks were accumulated in the range of 190–400 nm, and the column temperature was set at 25°C.

### 2.3. Mice and Virus

Male BALB/c mice, 5 to 6 weeks old, were purchased from Shanghai SLAC Laboratory Animal Co. Ltd. (Certificate number 2007000548167, SCXK (Hu) 2012-0002; Shanghai, China) and bred and maintained in a closed breeding facility at the Animal Center of Fudan University. All animal experiments were performed in accordance with the guidelines of the Ethics Committee for Animal Use of Fudan University. The mouse adapted influenza A/FM/1/47 (H1N1) virus was provided by the Institute of Medicinal Biotechnology, Chinese Academy of Medical Sciences (Beijing, China), and stored at −80°C until use. Mice were infected intranasally (i.n.) with 10 LD_50_ doses of H1N1 virus in 1640 culture medium.

### 2.4. Experimental Design

**Figure 2:**
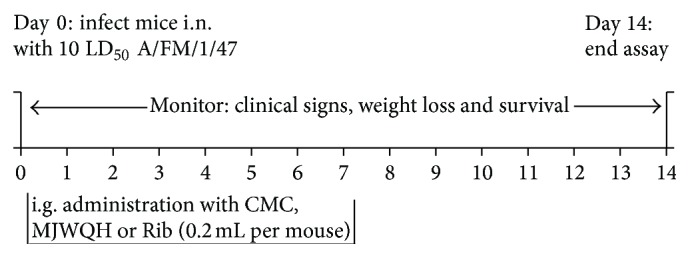
Experimental protocol. All BALB/c mice except the NC ones were challenged intranasally with influenza A/FM/1/47 (H1N1) virus (10 LD_50_ per mouse) and then treated with 0.5% CMC solution (model control, MC), MJWQH, or Rib one hour later for 2, 4, or 7 days, respectively.

The BALB/c mice were randomly divided into four groups: normal control (NC); model control (MC); MJWQH; and ribavirin (Rib). The ribavirin was supplied by Star Lake Bioscience Co., Inc. (Guangdong, China).  Figure 2 illustrates our initial protocol. On day 0, BALB/c mice (except for the NC) were anesthetized with isoflurane and infected i.n. with 10 LD_50_ doses of influenza A/FM/1/47 H1N1 virus in a volume of 30 *μ*L. One hour later, the MJWQH and Rib mice were intragastrically (i.g.) administered with MJWQH (1.8 g/kg/d) or ribavirin (100 mg/kg/d dissolved in 0.5% carboxyl methyl cellulose (CMC) solution). The NC and MC mice were treated with the same volume of 0.5% CMC solution. All mice were monitored daily for clinical symptoms, body weight, and survival for 14 consecutive days [2]. Mice showing more than 25% of body weight loss were considered to have reached the experimental endpoint and were then humanely euthanized by CO_2_ asphyxiation.

### 2.5. Pathological Examination and Immunohistochemistry

Lungs were removed on day 4 after infection, weighed, and then inflated with 10% phosphate-buffered formalin to their normal volume. The lung index (ratio of lung weight to body weight) was calculated as a parameter of lung edema. The left lobes were paraffin embedded and cut into 5 *μ*m thick sections, and one section from each tissue sample was stained using a standard haematoxylin-and-eosin (HE) procedure. Lung sections were examined to determine the extent of pneumonia in a blinded fashion as previously described [18]: 0 = no pneumonia; 1 = mild interstitial pneumonia (<25% of the lung); 2 = moderate interstitial pneumonia (25–50% of the lung); and 3 = severe interstitial pneumonia (>50% of the lung). The scores of the individual samples were summed up to yield a composite score.

The tissue sections were immunostained using the streptavidin-biotin-horseradish peroxidase method. The sections were deparaffinized and rehydrated through a graded series of alcohols and then microwaved in EDTA buffer (pH 8.0) at 97°C for 12 min to unmask antigen epitopes. The sections were treated with 3% hydrogen peroxide methanol solution for 10 min to block endogenous peroxidase, washed in PBS, and incubated with rabbit anti-NF-*κ*B p65 antibody (Assay Biotech, USA) at 1 : 200 dilution. They were washed again in PBS and incubated with avidin-biotin complex-horseradish peroxidase for 1 h at room temperature. The sections were incubated with chromogen-fast diaminobenzidine (DAB) for 1–5 min, after which they were counterstained in haematoxylin and mounted on aqueous mounting medium.

### 2.6. Hemagglutination (HA) Test

Lung tissues pooled from all mice in each group were homogenized in 1 mL of sterilized PBS. The homogenates were centrifuged at 10000 ×g for 10 min, and the resulting supernatant was analyzed by HA assay. Serial 2-fold dilutions of each viral preparation were made in PBS (pH 7.15) in 96-well V-bottomed microplates, and 25 *μ*L of 0.5% suspension of chicken red blood cells (RBCs) was added to each well. The contents in plates were mixed and incubated at room temperature (22–25°C) until complete agglutination of erythrocytes. The HA titer was the reciprocal of the dilution of virus in the last well with complete hemagglutination.

### 2.7. ELISA

Lungs were homogenized in PBS and the supernatants were collected. The inflammatory cytokines (IL-1, IL-6, TNF-*α*, and IFN-*α*) and chemokines (MCP-1, RANTES, MIP-1, and IP-10) in the supernatants of each group were tested by ELISA kits (Abcam, England) supplied by Jinma Laboratory Equipment Co., Ltd. (Lot number: 201303; Shanghai, China), which was performed according to the manufacturer's instructions. ELISAs were read on a 96-well plate reader and concentrations were determined using Revelation software (BioTek Epoch, USA).

### 2.8. Western Blotting

Lung protein extracts were prepared using the Nuclear Extract Kit (BestBio, China) following the manufacturer's instruction. Equal amounts of protein were separated by SDS-PAGE and subsequently blotted on polyvinylidene fluoride membranes (220 mA, 65 min). Blots were blocked in TBS solution containing 0.1% Tween 20 and 5% nonfat dry milk overnight at 4°C. The following antibodies and dilutions were used: TLR7 (Catalog number 2633, Cell Signaling Technology; 1 : 1000); MyD88 (D80F5) (Catalog number 4283, Cell Signaling Technology; 1 : 1000); TRAF6 (Catalog number 04-451, Millipore, USA; 1 : 1000); I*κ*B-*α* (c-21) (sc-371, Santa Cruz Biotechnology, 1 : 200); p-IKK*α*/*β* (Ser 176) (sc-21661, Santa Cruz Biotechnology, 1 : 200); NF-*κ*B p65 (sc-109, Santa Cruz Biotechnology, 1 : 200); p-NF-*κ*B p65 (Ser 276) (sc-101749, Santa Cruz Biotechnology, 1 : 200); and GAPDH (14C10) (Catalog number 2118, Cell Signaling Technology; 1 : 1000). The membranes were blotted with appropriate secondary antibodies (Immunology Consultants Laboratory, Inc., USA; 1 : 5000), and the blotted proteins were visualized by enhanced chemiluminescence using a commercially available kit (Millipore, USA).

### 2.9. Real-Time Reverse Transcription-PCR

Lungs were homogenized with a homogenizer to prepare the suspension, which was serially diluted in Trizol reagent (1 mL of Trizol for 100 mg tissue). Total RNA was prepared using the RNA fast isolation kit (Shanghai Generay Biotech Co., Ltd., Shanghai, China). For first-strand cDNA synthesis, 1 *μ*g of total RNA was primed with random primers by reverse transcriptase (Promega, USA), and then 1/20 volume of cDNA was amplified on a StepOne Plus instrument using SYBR Green Real-Time PCR Master Mix Reagent (Toyobo, Japan). The PCR was performed at 95°C for 10 min, followed by 35 cycles of 95°C for 10 s, 58°C for 15 s, and 72°C for 30 s. Amplification was followed by melting curve analysis. The primers were designed with Beacon Designer 7 software (Table 2). The level of gene transcription was determined by comparing with the NC group.

### 2.10. Statistical Analyses

All statistical analyses were performed using GraphPad Prism 5.0 software (GraphPad Software, Inc., CA, USA). Survival curves were estimated by the Kaplan-Meier method and their homogeneity was estimated by the log-rank test. Multiple group comparisons were performed using one-way analysis of variance (ANOVA), followed by Dunnett's test to determine significant differences from the control. A *P* value less than 0.05 was considered significant for all tests.

## 3. Results

### 3.1. MJWQH Increased the Survival Rate of the H1N1-Infected Mice

**Figure 3:**
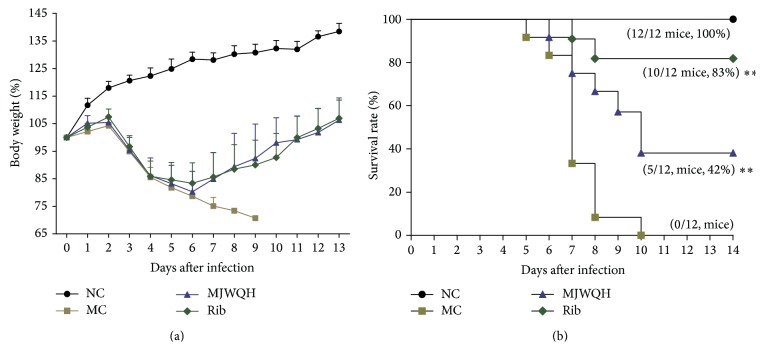
MJWQH treatment protected mice from lethal influenza challenge. BALB/c mice (*n* = 12 mice/group) were treated as described in Figure 2 for 7 days and monitored daily for signs and symptoms, body weight, and survival for 14 consecutive days. (a) Body weight (means ± SEM); (b) survival (number of survivors/total number of mice).  ^**^ *P* < 0.01 versus the MC group.

Mice receiving 0.5% CMC developed the most severe symptoms, that is, lethargy, ruffled fur, hunched posture, piloerection, rapid shallow breathing, and audible rattling, whereas some MJWQH and Rib treated mice presented such symptoms over the 14 days of treatment. The CMC-treated mice showed a progressive weight loss from day 3 after infection onward (Figure 3(a)), while the MJWQH and Rib treated mice showed a comparable weight loss from day 3 through day 6 after infection, but followed by a steady weight gain. The survival curves further confirmed the efficacy of MJWQH against the lethal influenza infection (Figure 3(b)). It showed that all MC mice died by day 10 after infection; the MJWQH treatment significantly increased the survival of H1N1-infected mice (42%; *P* < 0.01 versus the MC group), and the Rib treatment further increased the survival rate up to 83% (*P* < 0.01 versus the MC group). The survival curves were ended on day 14 after infection as no further mortality occurred after this time point.

### 3.2. MJWQH Alleviated the Severity of Lung Injuries

**Figure 4:**
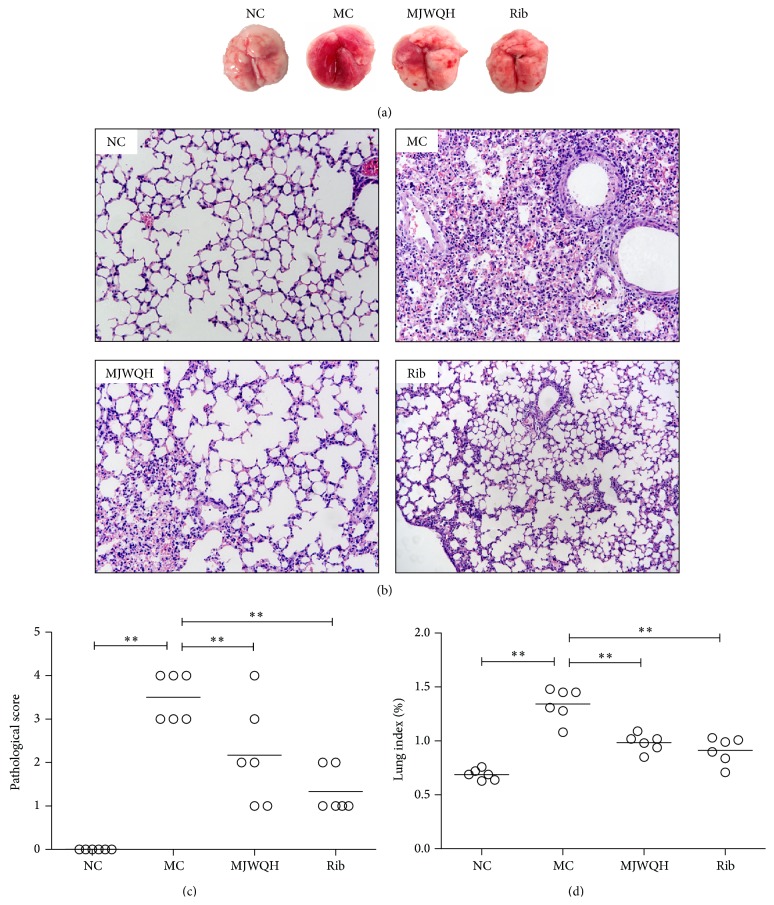
MJWQH alleviated the severity of H1N1-induced lung injuries. BALB/c mice (*n* = 6 mice/group) were treated as described in Figure 2 for 4 days. (a) Macroscopic appearance of lungs; (b) pathological changes of lung tissues (HE, ×200); (c) pathological scores; and (d) lung index. Data were presented as mean ± SD.  ^**^ *P* < 0.01 versus the MC group.

Lungs were removed on day 4 after infection for gross observation and histopathological examination. Edema, consolidation, and profuse hemorrhage were observed in the MC mice, whereas significantly less lung damage was observed in the Rib or MJWQH treated mice (Figure 4(a)). Histopathological analysis also revealed a significant reduction in the thickness and congestion of alveolar walls, intra-alveolar edema, and infiltrated neutrophils in lung tissues of mice treated with Rib and MJWQH (Figure 4(b)). In agreement with these histopathological findings, the Rib and MJWQH treated mice had significantly lower pathological scores (Figure 4(c)) and lung index (Figure 4(d)) than the MC group on day 4 after infection. In summary, MJWQH treated mice had a reduced lung injury after a lethal inoculum of pandemic H1N1 influenza virus.

### 3.3. MJWQH Inhibited Influenza Virus Replication* In Vivo*

**Figure 5:**
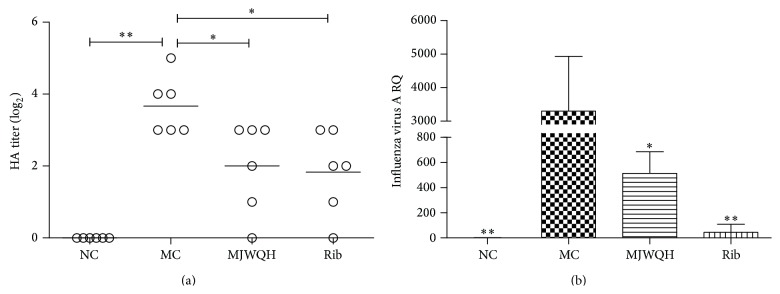
MJWQH inhibited virus replication. BALB/c mice (*n* = 6 mice/group) were treated as described in Figure 2 for 4 days. (a) HA titers of lung homogenates; (b) relative quantitation of influenza A virus. Data were presented as mean ± SD.  ^*^ *P* < 0.05;  ^**^ *P* < 0.01 versus the MC group.

The antiviral effect of MJWQH was determined by HA titers and the relative quantitation (RQ) of influenza virus replication in the lungs. By day 4 after infection, the viral titer was increased in all infected mice as compared to the NC group (Figure 5(a)). However, MJWQH or Rib significantly inhibited virus replication, as evidenced by a significant reduction in HA titer (*P* < 0.01 versus the MC group, Figure 5(a)) and the RQ of influenza A virus in the lungs (*P* < 0.05 versus the MC group, Figure 5(b)), indicating that both of them were effective in decreasing the viral load in mice infected by H1N1.

### 3.4. MJWQH Suppressed the Secretion of Cytokine/Chemokine Induced by H1N1 Influenza Virus

**Figure 6:**
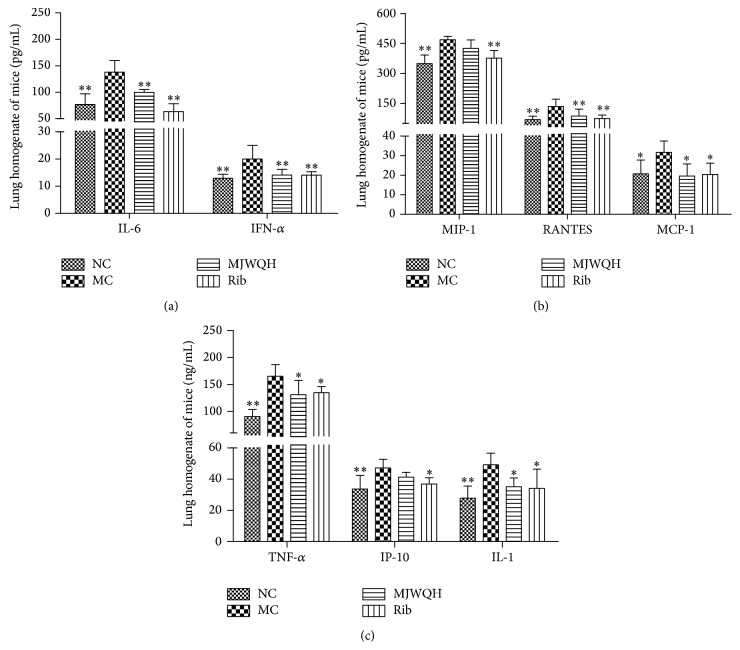
MJWQH suppressed innate immune responses. Proinflammatory cytokine and chemokine levels were analyzed in lung homogenates of mice on day 4 after infection by ELISA. Data were presented as mean ± SD.  ^*^ *P* < 0.05;  ^**^ *P* < 0.01 versus the MC group.

Early dysregulated innate immune responses in the lung are associated with morbidity and mortality during infection with highly pathogenic strains of influenza virus [8, 19]. Robust innate proinflammatory cytokine expression is believed to cause direct tissue insult and to recruit potentially tissue destructive inflammatory cells. The results showed significant reductions in TNF-*α*, IL-1, IL-6, MCP-1, RANTES, and IFN-*α* in the MJWQH treated mice on day 4 after infection. However, in comparison to the Rib treatment, MJWQH failed to suppress MIP-1 and IP-10 on day 4 after infection (Figures 6(a)–6(c)). All these results indicated that MJWQH suppressed cytokine production in severe pneumonia.

### 3.5. MJWQH Downregulated TLR7-MyD88-Dependent NF-*κ*B Signaling Pathway

**Figure 7:**
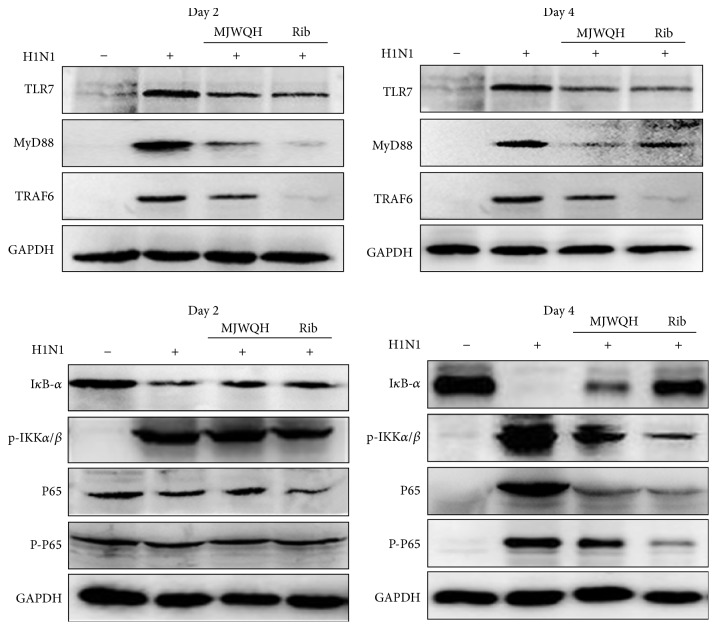
MJWQH inhibited the NF-*κ*B signaling pathway. BALB/c mice (*n* = 6 mice/group) were treated as described in Figure 2 and killed on day 2 or 4 after infection. Cytosolic fractions and nuclear extracts were prepared from lung homogenates. NF-*κ*B p65, I*κ*B-*α*, TLR7, MyD88, and TRAF6 levels were detected by specific antibodies in cytosolic fractions using Western blot analysis. Phosphorylation of IKK*α*/*β* and p65 was determined in the nuclear extracts. GAPDH protein was used as a loading control.

NF-*κ*B is an important transcription factor for the induction of various inflammation-associated genes, including cytokines and chemokines. To better understand the therapeutic mechanism of MJWQH in mice infected with H1N1 virus, we measured the expressions of TLR7, MyD88, TRAF6, p65, p-p65, I*κ*B-*α*, and p-IKK*α*/*β* in lung tissues on days 2 and 4 after infection by using Western blot analysis.  Figure 7 showed that influenza H1N1 infection significantly increased the protein expressions of TLR7, MyD88, and TRAF6 on days 2 and 4 after infection, which could be downregulated by the Rib or MJWQH treatment. We then examined whether MJWQH regulated the nuclear translocation of NF-*κ*B as well as the phosphorylation and proteolytic degradation of I*κ*B-*α*. No effect was observed on the NF-*κ*B signaling pathway on day 2 after infection. However, NF-*κ*B subunit p65 and p-p65 proteins in nucleus were significantly increased in virus-infected mice on 4 day after infection, but this increase could be suppressed by the MJWQH or Rib treatment. MJWQH also significantly inhibited the proteolytic degradation of I*κ*B-*α* in mice. IKK*α*/*β* was expected to make an essential contribution to I*κ*B phosphorylation, so we examined the p-IKK*α*/*β* level. The results showed that MJWQH significantly inhibited p-IKK*α*/*β*. These results together suggested that MJWQH inhibited H1N1 infection by inhibiting NF-*κ*B signaling pathway on day 4 after infection.

**Figure 8:**
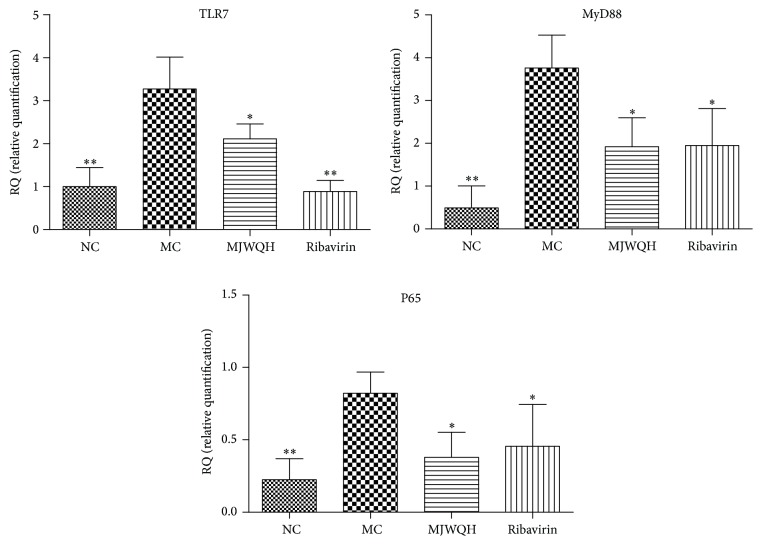
MJWQH inhibited the NF-*κ*B signaling pathway. BALB/c mice (*n* = 6 mice/group) were treated as described in Figure 2 and killed on day 4 after infection. Lungs were processed for total RNA and subjected to real-time PCR for detection of TLR7, MyD88, and NF-*κ*B p65 gene expressions, using GAPDH as a housekeeping gene. Data were presented as mean ± SD.  ^*^ *P* < 0.05;  ^**^ *P* < 0.01 versus the MC group.

To further demonstrate that MJWQH inhibited the NF-*κ*B signaling pathway in the RNA level, we quantified the mRNA levels of TLR7, MyD88, and p65 in lung tissues on day 4 after infection.  Figure 8 showed that all of them were significantly reduced in the Rib and MJWQH treated mice.

**Figure 9:**
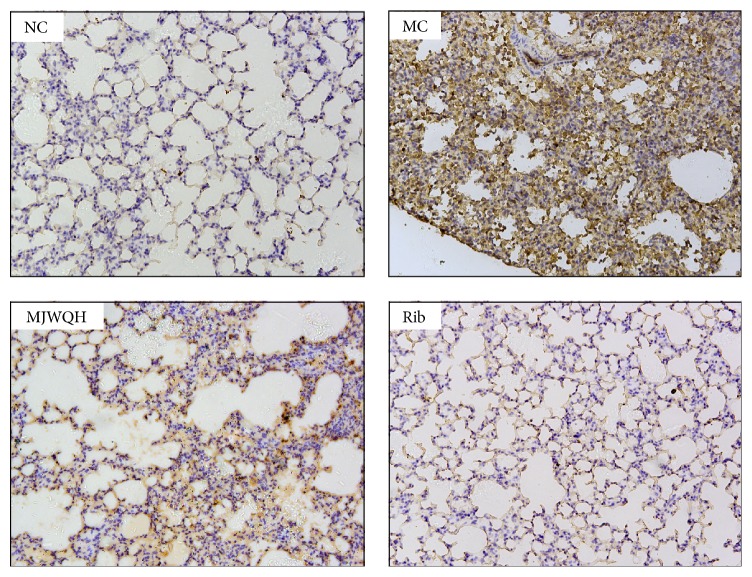
MJWQH inhibited the NF-*κ*B expression in lung tissue (×200). BALB/c mice (*n* = 6 mice/group) were treated as described in Figure 2 and killed on day 4 after infection. Lung sections were stained with rabbit anti-p65 (brown) and counterstained with hematoxylin.

Finally, we examined NF-*κ*B signaling in lungs of H1N1-infected mice using immunohistochemistry with antibodies against p65 on day 4 after infection.  Figure 9 showed that p65 could be hardly detected in the NC mice but it was abundant in the lungs of MC mice. However, there was a significant lower expression of p65 in the lungs of the Rib and MJWQH treated mice.

All these results clearly demonstrated that MJWQH blocked influenza virus infection by inhibiting the activation of TLR7/MyD88/TRAF6/IKK*α*/*β*/NF-*κ*B signaling pathway.

## 4. Discussion

MJWQH is composed of eight herbs, each having specific antiviral, anti-inflammatory, or antioxidant activities.* Radix Scutellariae* has inhibitory effects on the influenza virus, hepatitis B virus, and human immunodeficiency virus [20–22];* Radix Notopterygii *has antioxidant and anti-inflammatory activities [23];* Herba Taraxaci* has antiviral, antiseptic, and anti-inflammatory activities [24];* Radix Astragali* has antioxidant, immunopotentiating, and antistress activities [25, 26];* Radix Saposhnikoviae* has immunoregulatory and antioxidant activities [27];* Rhizoma Atractylodis* has potent antiviral and antioxidant activities [28];* Herba Houttuyniae* has inhibitory effects on acute inflammation [29, 30]; and* Radix Trichosanthis *has antiviral activities and inhibits the replication of human immunodeficiency virus type 1 (HIV-1) [31, 32]. These biological activities would be helpful for severe pneumonia induced by influenza virus. However, the mechanism of their combined actions has not yet been verified.

A/FM/1/47 is a mouse-adapted influenza virus which exhibits high virulence and can induce strong immune responses and inflammation in the murine lung [33]. The effect of MJWQH on the H1N1-induced severe pneumonia was evaluated in a murine acute lung injury model in this study. The results showed that MJWQH significantly relieved the signs and symptoms, reduced body weight loss, and improved the survival rate of H1N1-infected mice. It also significantly inhibited virus replication, as evidenced by a significant reduction in HA titer and the relative quantitation of influenza A virus in the lungs. Altogether, these results suggested that MJWQH had potent antiviral activity against H1N1 influenza A virus infection in mice. Our previous studies showed that there was no reduction in the virus titer in the A549 cells infected by influenza A virus at 24 hours after infection (data not shown here). However, in this study, we found that the MJWQH treatment significantly reduced lung HA titers. Therefore, we speculated that the antiviral activity of MJWQH was accomplished by inhibiting the early recruitment of inflammatory leukocytes to the lungs and suppressing excessive innate inflammatory responses.

Highly pathogenic flu can trigger excessive immune response or cytokine storm, which in turn leads to immune damage to the lung. In the case of infection, inflammation begins when the cells of the innate immune system recognize a pathogen-associated molecular pattern; then, certain host cells begin to secrete chemokines. Chemokines are small proteins (<10 kDa) that can activate and mediate the migration of leukocytes to the site of infection or inflammation [34]. Cytokines can be secreted by a variety of cells, including phagocytic cells such as macrophages and neutrophils, despite the fact that the endothelial cells are responsible for over half of all produced various cytokines and chemokines during inflammatory processes. [35]. The early induction of cytokines and chemokines is associated with symptom formation in humans [36–38]. IFN-*α* activates inflammatory cells and stimulates expression of multiple cytokines and chemokines [39–41]. TNF-*α*, IL-1, and IL-6 have multifunctional activities and are associated with morbidity during influenza virus infection. Chemokines such as MCP-1, MIP-1, RANTES, and IP-10 induce the recruitment of innate immune cells into the lung, which can release more cytokines exacerbating cytokine storm and further damage the lung [42–44]. Therefore, balancing the inflammatory network may represent an effective treatment for influenza virus infection. We demonstrated in this study that the production of proinflammatory cytokines and chemokines from lung tissues following influenza infection was regulated by MJWQH. Thus, the efficacy of MJWQH is likely due to its ability to inhibit excessive innate immune response and immune-mediated pulmonary tissue injury.

TLRs are key receptors in innate immune recognition and play an important role in the initiation of acquired immune response [45]. TLR7 has been demonstrated to be an intracellular receptor that recognizes ssRNA after the ribonucleoprotein complex has been degraded inside acidified endosomes [46, 47] and triggers cytokine secretion through the adapter MyD88, which recruits signaling mediators that activate NF-*κ*B [48]. NF-*κ*B could regulate the gene transcription of growth factors, transcription factors, cytokines, chemokines, and interferons [49]. Recognition and rapid clearance of pathogens by the innate immune system provide the first line of defense against infection. Overexpression of cytokines and chemokines induced by influenza virus infection is dependent on NF-*κ*B signaling pathway. It has been well documented that inhibition of NF-*κ*B or proteasome leads to I*κ*B accumulation and inhibits influenza virus replication* in vitro* and* in vivo* [50–52]. The signaling cascade of all activated TLRs starts with the activation of MyD88, which activates TRAF6 and eventually leads to the activation of NF-*κ*B [53]. MyD88 is a crucial adaptor protein downstream of TLRs, whereas TRAF6 is a signal transducer in the NF-*κ*B pathway, both of which are essential for the production of inflammatory cytokines [54–56]. IKK stimulates NF-*κ*B activity by causing phosphorylation and degradation of I*κ*B proteins, the cytoplasmic inhibitors of NF-*κ*B [57]. Activated IKK promotes the dissociation of the cytosolic inactive NF-*κ*B-I*κ*B complexes via the serine phosphorylation and degradation of I*κ*B, leading to NF-*κ*B translocation to the nucleus and transcriptional upregulation of inflammatory genes [58]. The inhibitory effect of MJWQH on the NF-*κ*B signaling pathway could be associated with the inhibition of IKK activation. We found that I*κ*B and p65 levels were not affected on day 2 after infection; but p65 and p-p65 expressions were significantly increased on day 4 after infection. The real time-PCR and immunohistochemistry analysis revealed similar results. Therefore, it can be concluded that MJWQH modulates the immune response by inhibiting the TLR7-MyD88-dependent NF-*κ*B signaling pathway. The antiviral effects of MJWQH might relate to the inhibition of TLR7/MyD88/TRAF6/IKK*α*/*β* NF-*κ*B signaling pathway. However, as MJWQH contains numerous compounds with various pharmacological activities, it is not clear which compounds are responsible for the immune regulation. This underscores the need to further determine whether MJWQH directly blocks the combination of virus and TLR7 and what the active components are and how they work.

Taken together, the present study showed that early MJWQH treatment contributed to recovery from severe pneumonia due to its anti-inflammatory activity through regulating TLR7/MyD88/NF-*κ*B signaling pathway. The results presented in this study may provide compelling evidence for the therapeutic potential of MJWQH as an alternative therapy to the western medicine in the treatment of influenza virus infections.

## Figures and Tables

**Table 1:** Composition of MJWQH.

Chinese name	Latin name	Amount (g)	Place of origin
Qiang Huo	Rhizoma et Radix Notopterygii	5	Inner Mongolia, China
Cang Zhu	Rhizoma Atractylodis	10	Sichuan, China
Pu Gong Yin	Herba Taraxaci	20	Jiangsu, China
Huang Qin	Radix Scutellariae	10	Inner Mongolia, China
Huang Qi	Radix Astragali	20	Gansu, China
Fang Feng	Radix Saposhnikoviae	5	Hebei, China
Yu Xing Cao	Herba Houttuyniae	10	Gansu, China
Tian Hua Fen	Radix Trichosanthis	15	Henan, China

**Table 2:** RT-PCR primer sequence.

Gene	Sequence
Influenza A virus M	
Forward	5′-GACCGATCCTGTCACCTCTGAC-3′
Reverse	5′-AGGGCATTCTGGACAAAGCGTCTA-3′
GAPDH	
Forward	5′-ACCACCATGGAGAAGGCTGG-3′
Reverse	5′-CTCAGTGTAGCCCAGGATGC-3′
TLR7	
Forward	5′-GGTGGCAAAATTGGAAGATCC-3′
Reverse	5′-AGCTGTATGCTCTGGGAAAGGTT-3′
MyD88	
Forward	5′-CCAGAGTGGAAAGCAGTGTC-3′
Reverse	5′-GTCCTTCTTCATCGCCTTGT-3′
TRIF	
Forward	5′-CCACGTCCTACACGGAAGAT-3′
Reverse	5′-AACAGCATCTGCAGCTACCA-3′
NF-*κ*B P65	
Forward	5′-ATGTGCATCGGCAAGTGG-3′
Reverse	5′-CAGAAGTTGAGTTTCGGGTAG-3′

## Abbreviations

BALF: Bronchoalveolar lavage fluid
IFN: Interferon
IL: Interleukin
TNF: Tumor necrosis factor
MCP-1: Macrophage chemotactic protein-1
RANTES: Regulated upon activation normal T cell expressed and presumably secreted
MIP-1: Macrophage inflammatory protein-1
IP-10: IFN-*γ*-inducible protein-10
NF-*κ*B: Nuclear factor kappa-light-chain-enhancer of activated B cells
TLR: Toll-like receptor
MyD88: Myeloid differentiation factor 88
TRAF6: Tumor necrosis factor receptor-associated factor 6
I*κ*B: Inhibitor of NF-*κ*B
IKK: I*κ*B kinase.
